# Error Rates in Race and Ethnicity Designation Across Large Pediatric Health Systems

**DOI:** 10.1001/jamanetworkopen.2024.31073

**Published:** 2024-09-03

**Authors:** Gary L. Freed, Brittany Bogan, Adam Nicholson, Deborah Niedbala, Susan Woolford

**Affiliations:** 1 Michigan Child Health Equity Collaborative, Ann Arbor; 2 Child Health Evaluation and Research Center, University of Michigan, Ann Arbor; 3 C. S. Mott Children’s Hospital and Von Voigtlander Women’s Hospital, Ann Arbor, Michigan; 4 Corewell Health Helen DeVos Children’s Hospital, Grand Rapids, Michigan; 5 Children’s Hospital of Michigan, Detroit

## Abstract

**Importance:**

Without knowledge of the degree of misattribution in racial and ethnic designations in data, studies run the risk of missing existing inequities and disparities and identifying others that do not exist. Further, accuracy of racial and ethnic designations is important to clinical care improvement efforts and health outcomes.

**Objective:**

To determine the error rate of racial and ethnic attribution in the electronic medical records (EMRs) across the 3 largest pediatric health systems in Michigan.

**Design, Setting, and Participants:**

This cross-sectional study collected race and ethnicity data from parents in outpatient clinics, emergency departments, and inpatient units at the 3 largest pediatric health systems in Michigan. A total of 1594 parents or guardians participated at health system A, 1537 at health system B, and 1202 at health system C from September 1, 2023, to January 31, 2024. Parent or guardian report of race and ethnicity for a child was used as the gold standard for comparison with the designation in the EMR.

**Exposure:**

Race and ethnicity designations in the EMR. Options for race designation across the health systems ranged from 6 to 49; options for ethnicity, from 2 to 10.

**Main Outcomes and Measures:**

Matching occurred in 3 stages. First, the exact racial and ethnic designations made by parents for their child were compared with what was found in the EMR. Second, for any child whose parent selected more than 1 racial category or for whom more than 1 appeared in the EMR, the designation of a minoritized racial group was used for matching purposes. Third, starting with the product of stage 2, racial designations were combined or collapsed into 6 (health systems A and C) or 5 (health system B) designations.

**Results:**

A total of 4333 survey responses were included in the analysis. The greatest error rate across the health systems occurred with the exact match of parental report of racial designation with the EMR, which ranged from 41% to 78% across the health systems. Improvement in the matching rate for each health system occurred with consolidation of race options provided. Differences between the health systems narrowed at the final consolidation to varying from 79% to 88% matching. Ethnicity matching between the EMR and the parental report ranged from 65% to 95% across the health systems. Missing race or ethnicity data in the EMR was counted as a nonmatch. Rates of missing racial data varied across the health systems from 2% to 10%. The health system with the greatest number of options for race and ethnicity had the highest error rates.

**Conclusions and Relevance:**

Although there will always be some misattribution of race and ethnicity in the EMR, the results of this cross-sectional study suggest that significant error in these data may undermine strategies to improve care. It is unclear whether those in an organization who determine the number of potential categories are the same persons who use those data to investigate potential disparities and inequities.

## Key Points

**Question:**

How accurate are racial and ethnic designations for children in electronic medical records (EMRs)?

**Findings:**

In this cross-sectional study of race and ethnicity data accuracy that included 4333 participants, rates of exact match of parental report of race with racial designation in the EMR ranged from 41% to 78% across 3 health systems. Rates of ethnicity matching between parental report and the EMR ranged from 65% to 95% across the health systems.

**Meaning:**

Error rates of these magnitudes raise doubts regarding data, suggesting either the presence or absence of inequities and disparities in specific areas of clinical care, and may undermine strategies to improve care.

## Teaser

This cross-sectional study evaluates the error rate of racial and ethnic attribution in the electronic medical records compared with parental report across the 3 largest pediatric health systems in Michigan.

## Introduction

Numerous studies have demonstrated health inequities or disparities in the outcomes, processes, or patient experiences in the care of adults and children.^1,2^ Many of these studies^3,4,5,6,7^ have focused on variation in care relative to the racial and/or ethnic characteristics of a sample or a population of patients. Inherent to the precision of the findings of these studies is a clear understanding of the mechanisms by which the racial and ethnic characteristics were determined and the degree of accuracy of those designations.^8,9^ This is true both for studies of large public or private datasets as well as studies using the electronic medical record (EMR) within a given health system, practice, or hospital.^10^ Without knowledge of the degree of misattribution in racial and ethnic designations, studies run the risk of missing existing inequities and disparities and identifying some that do not exist. Further, accuracy of racial and ethnic designations is important to clinical care improvement efforts and health outcomes.

Further complicating this issue is the range in the number of potential categories used by health systems for racial and ethnic attribution. Some health systems have chosen consistency with the US Census Bureau, while others have expanded the choices available for patients as part of inclusivity programs.

Another vexing issue has been the proportion of missing data for race and ethnicity in some data sources. Investigators have either removed individuals with missing data or used a variety of strategies to account for them; the degree of veracity and the impact of those efforts is highly variable.^11,12,13^ Further, and potentially more importantly, verification of the degree of accuracy of racial and ethnic attribution in secondary data or EMRs is rarely undertaken,^14^ especially for children,^2^ raising potential validity issues regarding the results of many published studies using these variables.^15,16,17,18^ This study sought to determine the error rate of racial and ethnic attribution in the EMRs across the 3 largest pediatric health systems in the state of Michigan.

## Methods

This study was determined to be exempt from review by the University of Michigan Medical School Institutional Review Board owing to its classification as a quality improvement project. Informed consent was not required. We followed the Strengthening the Reporting of Observational Studies in Epidemiology (STROBE) reporting guideline.

### Data Collection

Each health system had a process in place for entry of race and ethnicity information at the time of initial registration as a patient and for verification at the time of check in for outpatient and/or inpatient care. However, none of the health systems had verified the regularity with which those processes were followed. We used parent or guardian report of race and ethnicity for the child as the gold standard for comparison with the designation in the EMR.

In the present study, for each health system (A, B, and C), the specific options for the classification of race and ethnicity available in the EMR were identified (Table 1). Health system A used 17 racial category options and 2 ethnicity options; health system B, 6 racial category options and 2 ethnicity options; and health system C, 49 racial category options and 10 ethnicity options. These options were then placed verbatim on printed data collection forms developed separately for each health system, resulting in 3 distinct data collection forms. Each health system used separate choices for race and ethnicity. The remainder of the forms provided identical wording describing the purpose of the study and requesting participation, as well a request to record their child’s name and birthdate. The forms were available in English, Spanish, and Arabic and were written at a sixth grade level.

**Table 1.** Classification of Race and Ethnicity in the EMR

Health system	Racial category options	Ethnic category options
A	American Indian or Alaska Native, Asian, Asian Indian, Black or African American, Chinese, Filipino, Guamanian or Chamorro, Japanese, Korean, Middle Eastern or North African, Native Hawaiian, Other Asian, Other Pacific Islander, Samoan, Vietnamese, White or Caucasian, and other	Hispanic and not Hispanic
B	American Indian or Alaska Native, Asian, Black or African American, Native Hawaiian or Other Pacific Islander, White or Caucasian, and other	Hispanic and not Hispanic
C	American Indian or Alaska Native, Asian, African American, American Indian, Arab, Asian Indian, Bahamian, Bangladeshi, Barbadian, Bhutanese, Black, Black or African American, Burmese, Cambodian, Chinese, Dominica Islander, Dominican, European, Filipino, Haitian, Hmong, Indonesian, Iwo Jiman, Jamaican, Japanese, Korean, Laotian, Madagascar, Malaysian, Maldivian, Melanesian, Micronesian, Middle Eastern or North African, Native Hawaiian or Pacific Islander, Nepalese, Okinawan, Other Pacific Islander, Pakistani, Polynesian, Singaporean, Sri Lankan, Taiwanese, Thai, Tobagoan, Trinidadian, Vietnamese, West Indian, White, and other	Central American, Cuban, Dominican, Hispanic or Latino, Latin American, Mexican, not Hispanic or Latino, Puerto Rican, South American, and Spaniard

Abbreviation: EMR, electronic medical record.

At each health system, parents were approached in a variety of outpatient clinics or the emergency department, either by a member of the research team or a staff member of the health system. They were requested to participate in a study to better understand the accuracy of racial and ethnic attribution in the EMR. Those who agreed to participate were provided with the form and an envelope in which to place the completed form and asked to select as many categories as they desired to categorize the race and ethnicity of their child. They were instructed to place the envelope in a designated box in the clinic or to return it to the person who provided it to them. Fewer than 5% of those approached at any site declined to participate. The data collection goal of the study was to achieve a convenience sample of 1200 to 1500 children at each health system.

Members of the research team then identified the demographic information for each child as recorded in the demographic section of the EMR of each health system and recorded it on the same data collection form completed by the parent or guardian. Data for both the parent report and the medical record were entered into a REDCap (research electronic data capture) database. Verification of accuracy of data entry was conducted for 5% of the sample, and no errors were found.

### Statistical Analysis

#### Data Analysis for Race Category Matching

Data analysis for matching of race category occurred in 3 stages. Stages were developed to determine the effect on the match rate by combining different racial categories in the parental report and the EMR.

In stage 1, the exact racial and ethnic designations made by parents or guardians for their child were compared with what was found in the EMR. A match required all designations to be the same in both the parental designation and the medical record. For example, if a parent selected both White and Black for racial designation, then both White and Black needed to be present in the medical record for a correct match to be designated.

In stage 2, for any child for whom the parent or guardian selected more than 1 racial category or for whom more than 1 appeared in the EMR, the designation of a minoritized racial group was used for matching purposes. When there was more than 1 minoritized racial group category selected, we prioritized designation for matching in the following order: Black or African American; American Indian, Alaska Native, or Pacific Islander; any Asian category used by the health system; Middle Eastern or North African; and other (eg, if the parent selected both White and Black, then in stage 2 the child was coded as of Black race). Other was a specific category for each health system that parents could choose and that appeared in the medical record.

In stage 3, starting with the product of stage 2, we then combined and collapsed racial designations into 6 (health systems A and C) or 5 (health system B) designations. The difference was due to health systems A and C having a separate Middle Eastern or North African designation that was not used by health system B. The 5 categories in common were American Indian or Alaska Native, Asian, Black or African American, White, and other. Matching was assessed to determine overall results from each of these 3 stages and, additionally for stage 3, the proportion of racial designations in the EMR that matched those provided by the parent or guardian in the survey and the proportion of racial designations provided by the parent or guardian in the survey that matched those found in the EMR.

#### Data Analysis for Ethnic Category Matching

For health systems A and B, the only ethnicity options in the medical record were Hispanic or non-Hispanic. Health system C had 10 ethnic response options (Table 1).

For health systems A and B, an absolute match rate was determined between parental report and the medical record. For health system C, an initial absolute match rate was conducted using all their ethnic designations. Subsequently, all ethnicity designations were collapsed to create a dichotomous selection between Hispanic and non-Hispanic, consistent with health systems A and B.

## Results

### Race Category Matching

**Figure 1.**
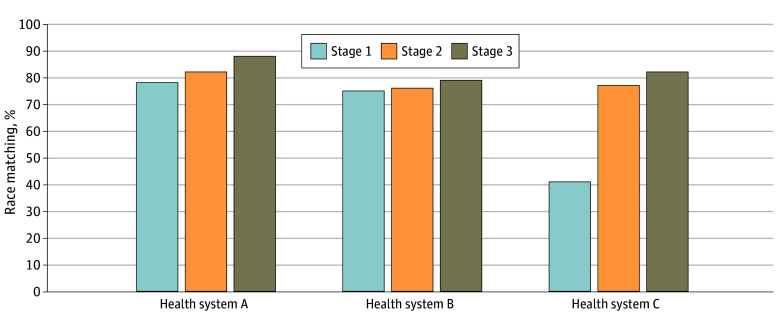
Electronic Medical Record and Survey Race Matching Percentage by Study Stage Across the 3 Health Systems

**Figure 2.**
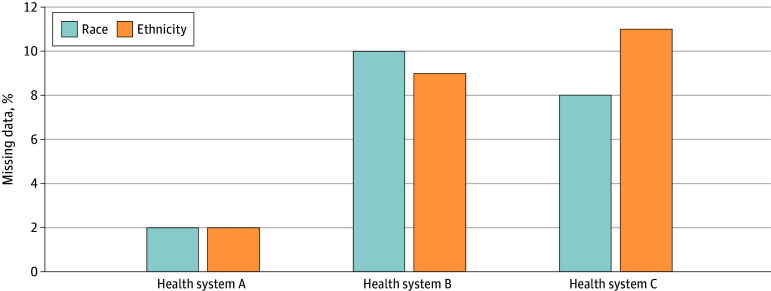
Missing Racial and Ethnic Data in the Electronic Medical Record Across the 3 Health Systems

A total of 4333 parents or guardians completed the surveys. There were 1594 parents or guardians who completed the survey at health system A, 1537 at Health system B, and 1202 at health system C. The greatest error rate across the health systems occurred with stage 1 matching, which ranged from 41% to 78% across the health systems. Improvement in the matching rate for each health system occurred with each subsequent matching stage. Differences between the health systems narrowed with stage 3 matching rates varying from 79% to 88% (Figure 1). Thus, in one health system, as many as 1 in 5 children were misidentified for race in the EMR and as many as 1 in 9 in the other two (Figure 2). Rates of missing racial data varied across the health systems from 2% to 10%.

Results of stage 3 racial matching for each health system are shown in Table 2. For each health system, there was error in the match rates across the racial designations. For example, regarding the accuracy of the designations in the EMR, for all health systems, at least 97% of those who were identified as Black in the EMR were also identified as Black on the survey. In contrast, for those who were identified as Black in the survey, the EMR contained a designation of Black ranging from 71% to 91% across the health systems. This indicated that the EMR undercounted children of Black race in all health systems but by varying degrees.

**Table 2.** Stage 3 EMR and Survey Race and Matching

Characteristic according to health system	Children’s racial designation by category, No.							EMR matches with survey, No. (%)
	American Indian or Pacific Islander	Asian	Black	Middle Eastern	White	Other	No data	
**Health system A (n = 1595)**								
No. of survey responses	25	122	345	51	989	48	14	NA
EMR data								
American Indian or Pacific Islander (n = 11)	8	1	0	0	2	0	0	8 (73)
Asian (n = 98)	2	96	0	0	0	0	0	96 (98)
Black (n = 317)	1	0	312	0	0	4	0	312 (98)
Middle Eastern (n = 10)	0	1	0	8	0	0	1	8 (80)
White (n = 1047)	11	12	21	33	949	16	5	949 (91)
Other (n = 85)	3	8	8	8	25	26	7	26 (31)
No data (n = 26)	0	4	4	2	13	2	1	1 (4)
Survey matches with EMR, No. (%)	8 (32)	96 (79)	312 (90)	8 (16)	949 (96)	26 (54)	1 (7)	1400 (88)
**Health system B (n = 1537)**								
No. of survey responses	33	63	265	NA	1045	100	31	NA
EMR data								
American Indian or Pacific Islander (n = 11)	8	1	1	NA	1	0	0	8 (73)
Asian (n = 35)	0	33	0	NA	0	2	0	33 (94)
Black (n = 193)	1	0	188	NA	0	3	1	188 (97)
Middle Eastern (NA)	NA	NA	NA	NA	NA	NA	NA	NA
White (n = 945)	11	2	16	NA	906	7	3	906 (96)
Other (n = 201)	10	13	38	NA	43	73	24	73 (36)
No data (n = 152)	3	14	22	NA	95	15	3	3 (2)
Survey matches with EMR, No. (%)	8 (24)	33 (52)	188 (71)	NA	906 (87)	73 (73)	3 (10)	1211 (79)
**Health system C (n = 1202)**								
No. of survey responses	7	43	854	62	177	38	21	NA
EMR data								
American Indian/Pacific Islander (n = 2)	0	1	1	0	0	0	0	0
Asian (n = 24)	1	19	2	2	0	0	0	19 (79)
Black (n = 799)	2	4	776	3	3	5	6	776 (97)
Middle Eastern (n = 42)	0	5	0	35	0	2	0	35 (83)
White (n = 172)	3	0	16	8	137	6	2	137 (80)
Other (n = 72)	0	8	12	6	23	17	6	17 (24)
No data (n = 91)	1	6	47	8	14	8	7	7 (8)
Survey matches with EMR, No. (%)	0	19 (44)	776 (91)	35 (56)	137 (77)	17 (45)	7 (33)	991 (82)

Abbreviations: EMR, electronic medical record; NA, not applicable.

For those identified as White in the EMR, surveys confirmed that designation between 80% and 96% across the health systems. For those who were identified as White in the survey, the EMR contained the same designation for those same children from 77% to 96% across the health systems.

### Ethnic Category Matching

Ethnic designation was completed by parents or guardians on the same survey form as for racial designation. Missing ethnicity data in the EMR were counted as a nonmatch. Rates of missing ethnicity data in the EMR varied from missing race data in the EMR across the health systems and ranged from 1% to 11% (Figure 2).

Ethnicity matching between the EMR and the survey ranged from 65% to 95% across the health systems. For those designated as Hispanic in the EMR, 68% to 94% of parents reported their children to be Hispanic, depending on the health systems. For those who were identified as Hispanic in the survey, the EMR contained a designation of Hispanic ranging from 33% to 81% of the time across the health systems (Table 3).

**Table 3.** EMR and Survey Ethnicity and Matching

Characteristic according to health system	Children’s ethnic designation by category, No.					EMR matches to survey, No. (%)	
	Hispanic	Non-Hispanic		No data (n = 0)			
**Health system A (n = 1594)**							
No. of survey responses	133	1424		37		NA	
EMR data							
Hispanic (n = 116)	108	4		4		108 (93)	
Non-Hispanic (n = 1456)	23	1400		33		1400 (96)	
No data (n = 22)	2	20		0		0	
Survey matches to EMR, No. (%)	108 (81)	1400 (98)		0		1508 (95)	
**Health system B (n = 1537)**							
No. of survey responses	247	1267		23		NA	
EMR data							
Hispanic (n = 208)	196	11		1		196 (94)	
Non-Hispanic (n = 1184)	31	1132		21		1132 (96)	
No data (n = 145)	20	124		1		1 (1)	
Survey Matches to EMR, No. (%)	196 (79)	1132 (89)		1 (4)		1329 (86)	
**Health system C (n = 1202)**							
No. of survey responses	203	758		241		NA	
EMR data							
Hispanic (n = 99)	67		19		13		67 (68)	
Non-Hispanic (n = 970)	109		673		188		673 (69)	
No data (n = 133)	27		66		40		40 (30)	
Survey matches to EMR, No. (%)	67 (33)		673 (89)		40 (17)		780 (65)	

Abbreviations: EMR, electronic medical record; NA, not applicable.

## Discussion

Among the most important findings from this study is the range of misattribution for race and ethnicity across the 3 health systems as well as the variability in the proportion of children with missing data. As similar comparative studies, to our knowledge, have not been conducted previously, it is unclear if these results represent a best- or a worse-case scenario. Regardless, they indicate a clear need for those who conduct studies to examine the presence of racial and ethnic disparities and inequities to assess the accuracy of the data used in those assessments.

As race and ethnicity are social constructs, there will never be an absolute gold standard for their designation in the EMR. However, if there is a desire or a societal imperative to assess the potential for disparities and inequities in care, some type of gold standard must be used to assess for accuracy in the EMR. The designation of a gold standard is inherently more complex for children than for adults. While adults can most often self-declare, children have the added complexity of the source of race and ethnicity designation used; race and ethnicity could be the mother, father, other caregiver, or, depending on age, the individual themselves (eg, if an adolescent). In this study, parental report was chosen as the gold standard.

Most previous studies of disparities and inequities using medical records, claims, or national datasets have not reported any assessments of either the accuracy of racial and ethnic designations or the rates of missing data within those data sources.^19,20^ Thus, the validity of such studies may be called into question. Depending on the degree of error in these variables, some of these studies may have missed disparities and inequities that exist and/or found some that do not. Either way, such occurrences would dilute true efforts to improve the care provided to children.^21^

Over the past several years, some health systems have expanded the number of categories available to patients for race and ethnicity to promote a sense of greater inclusivity. Although laudable, our data indicate that the greater the number of categories, the greater the potential for error in the designation of race and ethnicity in the EMR.^22^ It is unclear whether those in an organization who determine the number of potential categories are the same persons who will use those data to investigate potential disparities and inequities. If not, we suggest greater coordination within an organization may help to balance the goal of inclusivity with the reality of the use of data collected. At the least, transparent and deliberate communication regarding the need to consolidate or group race and ethnicity data for accurate analyses should occur. These efforts should also account for the White House Office of Management and Budget’s statistical policy directive Standards for Maintaining, Collecting, and Presenting Federal Data on Race and Ethnicity.^23^ This directive, announced in March 2024, provides up to 18 months for federal agencies to develop action plans regarding the incorporation of several changes to the way race and ethnicity data are collected. These changes include using one combined question for race and ethnicity and allowing respondents to select as many options as ally with how they identify, and the inclusion of a category of Middle Eastern or North African. Also of note, state and local jurisdictions may provide different guidance than federal authorities.

There are also complexities involved for health systems when attempting to improve the accuracy of race and ethnicity in the EMR. Depending on the health system, the origin of the EMR designations can be from a variety of sources, including registration personnel, self-service patient portal use, or clinic or hospital check-in staff.^24^ Often there are protocols in place for entering race and ethnicity into the medical record that may or may not be followed. For example, health system staff may be uncomfortable asking about race and ethnicity and simply enter their best assessment of the child. Increased training of health system personnel may be required to improve consistency and accuracy of such information placed into the EMR.

### Limitations

This study has some limitations. Parents were only offered the opportunity to complete the form in English, Spanish, or Arabic. Those who were only fluent in a different language would have been excluded. However, there are not large populations in the catchment area of these 3 hospitals that do not speak one of these 3 languages. Thus, we believe the impact on the study results is small.

Although the size of the sample provides face validity for verification of race and ethnicity data, the sample was not obtained in a randomized manner. Thus, results cannot be used to reflect the exact racial and ethnic distribution of the patient populations at each health system. Additionally, our use of parental or guardian report as the gold standard may have varied depending on the parent or guardian present with the child. If a different parent or guardian had accompanied the child, they may have completed the survey differently. Finally, we are unable to determine whether the error varies by the age of the child at the time of initial registration.

## Conclusions

This cross-sectional study found a range of misattribution for race and ethnicity and variability in the proportion of children with missing data. Without assessment of racial and ethnic disparities and inequities, efforts to measure improvement in health care for marginalized populations cannot occur. Although there will always be some misattribution of race and ethnicity in the EMR, significant error in these data may undermine strategies to improve care and raise doubts regarding studies that previously identified inequities and disparities. Health care systems should make efforts to assess the fidelity of their data and then ensure the data available are as accurate as possible. Further, once an understanding of the causes of missing data and misattribution are determined, attempts should be made to develop statistical correction factors to evaluate previous studies of disparities and inequities using those data and to account for discrepancies while new measurement and documentation strategies are established.

## Notes

Supplement 1.
Data Sharing Statement
