# Supplementary material for: Error Rates in Race and Ethnicity Designation Across Large Pediatric Health Systems
Source: JAMA Netw Open. 2024 Sep 3;7(9):e2431073. doi: 10.1001/jamanetworkopen.2024.31073 (PMC11372483; doi:10.1001/jamanetworkopen.2024.31073)
Supplement: Supplement 1. — Data Sharing Statement [file jamanetwopen-e2431073-s001.pdf]

Data Sharing Statement

Freed. Error Rates in Race and Ethnicity Designation Across Large Pediatric Health Systems. *JAMA Netw Open*. Published September 03, 2024. doi:10.1001/jamanetworkopen.2024.31073

Data
Data available: No

Additional Information
Explanation for why data not available: I, Gary Freed had full access to all the data in the study and takes responsibility for the integrity of the data and the accuracy of the data analysis. All information and materials in the manuscript are original.
